# Electrocardiographic Patterns as Predictors of Mortality in Aluminum Phosphide Poisoning: A Retrospective Cohort Single-Center Study

**DOI:** 10.7759/cureus.81713

**Published:** 2025-04-04

**Authors:** Noorul Hadi, Asfandiyar, Amna Saleem, Ashraf, Rumman, Zakir Ullah, Mohammad Hashim Khan

**Affiliations:** 1 Cardiology, Medical Teaching Institution Mardan Medical Complex, Mardan, PAK; 2 Research and Development, Pro-Gene Diagnostics and Research Laboratory, Mardan, PAK; 3 Pulmonology, Medical Teaching Institution Mardan Medical Complex, Mardan, PAK; 4 Pharmacovigilance/Active Drug Safety Monitoring and Management, Association for Community Development, Peshawar, PAK; 5 Cardiology, Mardan Medical Complex, Peshawar, PAK; 6 Cardiology, Khyber Medical University, Mardan, PAK

**Keywords:** aluminum phosphide poisoning, cardiotoxicity, electrocardiographic changes, phosphine toxicity, predicting mortality

## Abstract

Background

Aluminum phosphide (ALP) poisoning, prevalent in developing countries, poses a significant public health concern due to its high lethality, especially when ingested. ALP releases phosphine gas, which induces severe cardiotoxic effects, manifesting through various electrocardiographic (ECG) abnormalities. These ECG patterns are critical in predicting mortality among ALP poisoning patients. With mortality rates often exceeding 70%, early identification of these ECG markers can be pivotal in guiding clinical interventions, improving patient outcomes, and reducing the fatality associated with ALP poisoning. This study seeks to develop a prognostic model that uses ECG findings to predict the risk of mortality, providing a vital tool in resource-limited settings for rapid risk stratification and treatment optimization.

Objective

This study aimed to develop a prognostic model that utilizes ECG findings to predict the risk of mortality in patients with ALP poisoning.

Methodology

This retrospective cohort study was conducted at the Mardan Medical Complex from December 2022 to June 2024. The study included 64 patients diagnosed with ALP poisoning who met the inclusion criteria, focusing on those with complete medical records and ECG data. Patients were categorized into survivors and non-survivors. Statistical analyses included t-tests for continuous variables, chi-squared tests for categorical variables, and logistic regression to identify significant predictors of mortality. ECG patterns were analyzed using a random forest classifier to determine the most predictive features, and Kaplan-Meier survival curves were used to assess survival probabilities stratified by ECG abnormalities. Interobserver variability in ECG interpretation was resolved by consensus.

Results

Among the 64 patients studied, 44 (68.75%) were non-survivors, and 20 (31.25%) were survivors. Significant differences were observed in key variables between these groups. Non-survivors had a higher prevalence of atrial fibrillation (70.45% vs. 65.00%; p=0.047) and atrial premature beats (29.55% vs. 0%; p=0.006) and exhibited longer QT intervals (544.32±40.66 ms vs. 420.0±15.89 ms; p<0.001) and QRS durations (137.5±31.1 ms vs. 120.68±41.06 ms; p=0.047). They also had lower Glasgow Coma Scale (GCS) scores (6.61±3.51 vs. 8.2±3.55; p=0.029) and shorter hospital stays (20.39±27.17 hours vs. 75.80±15.00 hours; p=0.032), reflecting rapid clinical decline. Non-survivors ingested a higher number of ALP pills (4.52±1.56 vs. 2.5±0.95; p=0.043). Kaplan-Meier survival curves and logistic regression analyses confirmed that atrial fibrillation, QT prolongation, and wide QRS complexes were the strongest predictors of mortality, highlighting the prognostic significance of these ECG patterns in ALP poisoning.

Conclusion

This study confirms that the number of ALP pills ingested is a key determinant of the severity of cardiotoxic effects, as reflected in specific ECG patterns such as QT prolongation and atrial fibrillation. Recognizing this dose-dependent relationship can improve clinical outcomes by guiding the intensity of monitoring and treatment strategies based on the quantity of ALP exposure.

## Introduction

Aluminum phosphide (ALP) is a widely used fumigant and pesticide, particularly prevalent in agricultural regions where it serves as a cost-effective method for grain preservation and pest control [1]. Despite its efficacy in these applications, ALP is notorious for its high toxicity to humans, particularly when ingested [2]. It is highly poisonous and a significant cause of severe poisoning in developing countries. In Asia, where it is widely available, people use it as a method of suicide, making it a significant public health concern [3]. The chemical's lethality is primarily due to its ability to release phosphine gas when it comes into contact with moisture, such as water or stomach acid [4]. Phosphine is a potent cellular toxin that disrupts oxidative phosphorylation by inhibiting cytochrome c oxidase, leading to cellular hypoxia, metabolic acidosis, and, ultimately, multi-organ failure [5]. The rapid and often irreversible nature of ALP poisoning makes it a significant public health concern, particularly in developing countries where it is easily accessible.

The clinical presentation of ALP poisoning is highly variable, ranging from mild symptoms such as nausea, vomiting, and abdominal pain to severe systemic manifestations, including hypotension, arrhythmias, and profound shock [3]. The cardiovascular system is particularly vulnerable, with ALP-induced cardiotoxicity leading to a spectrum of electrocardiographic (ECG) abnormalities [6].

Mortality rates associated with ALP poisoning are alarmingly high, often exceeding 70% in severe cases [7]. The absence of a specific antidote exacerbates the situation, as treatment is largely supportive and focused on stabilizing the patient's hemodynamic status. Interventions typically include the administration of vasopressors, correction of electrolyte imbalances, and supportive care in an intensive care setting [3]. However, even with aggressive management, the prognosis remains poor, particularly in patients who present with severe ECG abnormalities and hemodynamic instability.

Given the critical need for early and accurate risk stratification, there is a growing interest in the use of ECG as a prognostic tool in ALP poisoning [8]. ECG is a readily available, non-invasive, and cost-effective diagnostic modality that can provide real-time insights into the cardiac status of the patient. Unlike other laboratory markers or imaging studies that may require specialized equipment or time-intensive processing, ECG can be performed at the bedside, making it particularly valuable in resource-limited settings where advanced diagnostic capabilities may be lacking.

In addition to the challenges of predicting mortality, there is also a need to understand the underlying pathophysiological mechanisms that link specific ECG patterns to poor outcomes. The cardiotoxic effects of ALP are believed to be multifactorial, involving direct myocardial injury, the disruption of ion channel function, and the induction of severe metabolic derangements such as acidosis and electrolyte imbalances [9,10]. These effects can manifest in a variety of ECG patterns, each of which may carry different prognostic implications. For example, QT prolongation is typically indicative of impaired myocardial repolarization, which can predispose patients to life-threatening arrhythmias like torsades de pointes. On the other hand, ST-segment elevation may reflect acute myocardial ischemia and infarction, both of which are associated with high mortality in the context of ALP poisoning [11,12].

The primary objective of this study is to develop a prognostic model that uses ECG findings to predict mortality risk in ALP poisoning patients. By analyzing the relationship between specific ECG patterns and patient outcomes, this study aims to identify key ECG markers associated with increased mortality. This model could enhance clinicians' ability to make informed decisions, especially in settings with limited diagnostic tools, and provide a basis for future research on targeted therapies for cardiac abnormalities linked to ALP poisoning. The model will consider ECG abnormalities along with clinical factors such as patient demographics, ALP dose ingested, and time since ingestion. This comprehensive approach aims to offer a valuable risk assessment tool, improving treatment decisions and understanding of phosphine gas cardiotoxicity, ultimately guiding the development of more effective treatments.

## Materials and methods

Study design

This was a retrospective cohort study conducted at Mardan Medical Complex from December 2022 to June 2024. After obtaining IRB approval, the sample was raised using a convenience sampling technique.

Inclusion criteria

We enrolled patients with a confirmed diagnosis of ALP poisoning based on clinical history and laboratory results positive for silver nitrate. Only individuals aged 18 years or older with complete medical records, including ECG data from admission through hospitalization, were considered. Our study specifically included patients who sought medical attention within 24 hours of ALP ingestion.

Exclusion criteria

Patients with pre-existing chronic cardiac conditions (such as diagnosed coronary artery disease or congenital heart defects) that could interfere with ECG interpretation were excluded. Additionally, cases with incomplete or missing ECG data were not included. Patients who had ingested other toxic substances or drugs that might influence ECG patterns were also excluded from the study.

Data collection

The study population was divided into survivors and non-survivors, with mortality as the primary outcome. Demographic, clinical, and ECG characteristics were compared between these groups using hospital records, encompassing patient demographics, clinical presentation, and outcomes. ECGs taken at admission, during hospitalization, and at critical points such as arrhythmia onset and clinical deterioration were analyzed. Demographic data included age, gender, body mass index (BMI), and medical history, while clinical data covered vital signs at admission, time from ingestion to hospital presentation, and Glasgow Coma Scale (GCS) score. The estimated ALP dosage was determined through inquiries with caregivers about the type of poisoning and the container in which the ALP tablets were found. The number of ingested pills was confirmed with patients at admission, with each tablet containing 3 g of dosage, consisting of 56.4% ALP and 44.6% ammonium carbonate. Patients were categorized into groups based on the number of tablets ingested: group 1 with one tablet (3 g), group 2 with two tablets (6 g), group 3 with three tablets (9 g), group 4 with four tablets (12 g), group 5 with five tablets (15 g), and group 6 with eight tablets (24 g) [3]. Interobserver variability was assessed, and any discrepancies were resolved by consensus between the cardiologists.

Statistical analysis

Data was analyzed using R Version 4.4.2 (R Foundation for Statistical Computing, Vienna, Austria (https://www.R-project.org/)). Descriptive statistics were utilized to summarize the demographic and clinical characteristics of the study population, with means and standard deviations for continuous variables and frequencies and percentages for categorical variables. Comparative analysis between survivors and non-survivors was performed using t-tests for continuous variables and chi-squared tests for categorical variables. Boxplots were employed to visualize the distribution of ECG patterns relative to GCS scores and estimated ALP pill ingestion, as well as to explore relationships between specific ECG patterns. A random forest classifier identified the most significant ECG features contributing to patient outcomes. Multidimensional scaling (MDS) was used to reduce the dimensionality of the ECG data, facilitating a two-dimensional visualization of the relationships between ECG patterns and ALP dosage. Decision tree analyses were conducted to predict key outcomes, with one tree focusing on GCS score prediction and another on the relationship between ALP dosage and ECG patterns. Kaplan-Meier survival curves compared survival probabilities for different ECG patterns using the log-rank test. Finally, logistic regression analysis assessed the association between various ECG patterns and the likelihood of non-survivor status. A p-value of <0.05 was considered statistically significant.

Ethical considerations

Ethical approval was obtained from the Ethical Committee of Medical Teaching Institution Bacha Khan Medical College (approval number: 632/BKMC). Given the retrospective nature of the study, the need for informed consent was waived, but confidentiality and privacy of patient data were strictly maintained.

## Results

Among the 64 patients analyzed, 44 (68.75%) were non-survivors, and 20 (31.25%) were survivors. No significant differences were observed in age, gender, BMI, body weight, or height between the groups. However, non-survivors exhibited significantly higher heart and respiratory rates both at admission and during hospitalization. They also ingested more ALP pills (4.52±1.56 vs. 2.5±0.95; p=0.043) and presented to the hospital sooner after ingestion (58.18±42.1 minutes vs. 73.0±76.37 minutes; p=0.008). Non-survivors had lower GCS scores (6.61±3.51 vs. 8.2±3.55; p=0.029) and shorter durations from admission to discharge. Symptomatically, non-survivors were more likely to present with diarrhea (72.73% vs. 70%), shortness of breath (70.45% vs. 65%), sweating (63.64% vs. 35%), abdominal pain (81.82% vs. 80%), and vomiting (90.91% vs. 90%), all of which were statistically significant. Other symptoms, such as drowsiness (54.55% vs. 50%), dyspepsia (45.45% vs. 40%), and cough (36.36% vs. 35%), did not show significant differences between the groups (Table 1).

**Table 1:** Comparison of demographic, clinical, and admission characteristics between survivors and non-survivors in patients with ALP poisoning P<0.05 is statistically significant. P-values with *** represent that no statistics were computed because the characteristic is present in either 100% or 0% of the population. BMI: body mass index; ALP: aluminum phosphide; GCS: Glasgow Coma Scale; kg: kilograms; mmHg: millimeters of mercury; bpm: beats per minute

Characteristics	Total (n=64)	Non-survivor (n=44)	Survivor (n=20)	P-values	Statistical tests	Test statistics
Age	22.57±5.92	23.59±7.3	21.55±4.55	0.111	t-value	1.612
Gender						
Male	27 (42.19%)	14 (31.82%)	13 (65%)	0.421	Chi-square	0.646
Female	37 (57.81%)	30 (68.18%)	7 (35%)			
BMI	25.91±6.39	25.45±7.83	24.37±6.96	0.435	t-value	0.783
Body weight (kg)	69.97±14.48	73.93±15.04	66.0±13.93	0.582	t-value	0.554
Height (feet)	5.45±0.48	5.47±0.52	5.42±0.44	0.103	t-value	0.651
At admission						
Diastolic pressure (mmHg)	66.09±9.03	50.68±8.73	81.5±9.33	0.702	t-value	1.195
Systolic pressure (mmHg)	92.95±9.14	68.41±8.61	117.5±9.67	0.411	t-value	1.619
Heart rate (bpm)	88.25±23.17	88.2±23.23	88.3±23.11	0.022	t-value	3.11
Respiratory rate (bpm)	22.12±4.18	23.23±5.02	21.0±3.34	0.017	t-value	2.014
Estimated ALP pill ingestion (count)	3.01±1.25	4.52±1.56	2.5±0.95	0.043	t-value	2.004
Time for ingestion to hospital admission (minutes)	65.59±59.23	58.18±42.1	73.0±76.37	0.008	t-value	2.564
During hospitalization						
Diastolic pressure (mmHg)	64.9±14.09	63.3±14.7	66.5±13.48	0.625	t-value	0.847
Systolic pressure (mmHg)	95.72±17.66	93.18±17.59	98.25±17.72	0.523	t-value	0.651
Heart rate (bpm)	97.44±19.55	98.23±21.96	96.65±17.13	0.043	t-value	2.356
Respiratory rate (bpm)	21.87±7.19	23.23±6.48	20.5±7.9	0.000	t-value	4.233
GCS	7.9±3.53	6.61±3.51	8.2±3.55	0.029	t-value	2.104
Admission to discharge time (hours)	37.70±35.24	20.39±27.17	75.80±15.00	0.032	t-value	3.206
Symptoms						
Diarrhea	46 (71.88%)	32 (72.73%)	14 (70%)	0.039	Chi-square	0.92
Drowsiness	47 (73.44%)	30 (68.18%)	17 (85%)	0.431	Chi-square	1.897
Dyspepsia	58 (90.63%)	39 (88.64%)	19 (95%)	0.493	Chi-square	0.74
Shortness of breath	44 (98.75%)	31 (70.45%)	13 (65%)	0.042	Chi-square	5.231
Sweating	35 (54.69%)	28 (63.64%)	7 (35%)	0.041	Chi-square	3.251
Cough	26 (40.63%)	17 (38.64%)	7 (45%)	0.892	Chi-square	0.057
Abdominal pain	52 (81.25%)	36 (81.82%)	16 (80%)	0.046	Chi-square	0.824
Vomiting	58 (90.63%)	40 (90.91%)	18 (90%)	0.048	Chi-square	0.15

Significant differences were observed in several ECG parameters between non-survivors and survivors. Atrial fibrillation was more prevalent in non-survivors (70.45% vs. 65%; p=0.047). Non-survivors also had higher incidences of atrial premature beats (29.55% vs. 0%; p=0.006) and U waves (29.55% vs. 0%; p=0.006). The Brugada pattern was noted in 36.36% of non-survivors compared to 45% of survivors (p=0.050). QT prolongation, ST-segment depression and elevation, and T-wave inversion were present in all non-survivors (100%) but absent in survivors, with highly significant p-values (p<0.001). Additionally, non-survivors exhibited longer PR intervals (121.23±12.92 ms vs. 113.2±29.03 ms; p=0.012), QRS durations (120.68±41.06 ms vs. 137.5±31.1 ms; p=0.047), and notably prolonged QT intervals (544.32±40.66 ms vs. 420.0±15.89 ms; p<0.001) (Table 2)

**Table 2:** Electrocardiographic characteristics and arrhythmic patterns in survivors and non-survivors of ALP poisoning P<0.05 is statistically significant. P-values with *** represent that no statistics were computed because the characteristic is present in either 100% or 0% of the population. ms: milliseconds; ALP: aluminum phosphide

Characteristics	Total (n=64)	Non-survivor (n=44)	Survivor (n=20)	P-values	Statistical tests	Test statistics
Total number of patients	64 (100)	44 (68.75)	20 (31.25)	***	***	***
Sinus rhythm	20 (31.25)	13 (29.55)	7 (35)	0.884	Chi-square	0.017
Atrial fibrillation	44 (68.75)	31 (70.45)	13 (65)	0.047	Chi-square	3.944
Atrial premature beats	13 (20.31)	13 (29.55)	0 (0)	0.006	Chi-square	7.612
Brugada pattern	16 (25)	16 (36.36)	9 (45)	0.050	Chi-square	3.45
Bundle branch block	25 (39.06)	16 (36.36)	9 (45)	0.478	Chi-square	0.504
Hyperkalemia arrhythmias	13 (20.31)	8 (18.18)	5 (25)	0.761	Chi-square	0.212
Left bundle branch block	25 (39.06)	14 (31.82)	8 (40)	0.059	Chi-square	0.019
Left ventricular hypertrophy	22 (34.38)	2 (4.55)	0 (0)	0.491	Chi-square	0.001
Low voltage QRS complexes	2 (68.75)	2 (4.55)	0 (0)	0.562	Chi-square	0.002
Pacemaker rhythm	22 (34.38)	14 (31.82)	8 (40)	0.056	Chi-square	1.154
QT prolongation	44 (68.75)	44 (100)	0 (0)	0.000	t-value	8.726
Right bundle branch block	25 (39.06)	16 (36.36)	9 (45)	0.041	Chi-square	0.054
Right ventricular hypertrophy	22 (34.38)	14 (31.82)	8 (40)	0.049	Chi-square	0.264
ST-segment depression	44 (68.75)	44 (100)	0 (0)	0.000	Chi-square	0.028
ST-segment elevation	44 (68.75)	44 (100)	0 (0)	0.000	Chi-square	1.122
T-wave inversion	44 (68.75)	44 (100)	0 (0)	0.000	Chi-square	2.556
U waves	13 (20.31)	13 (29.55)	0 (0)	0.006	Chi-square	3.122
Ventricular premature beats	25 (39.06)	16 (36.36)	9 (45)	0.433	Chi-square	0.543
Wide QRS complexes	25 (39.06)	16 (36.36)	9 (45)	0.478	Chi-square	0.264
PR interval duration (ms)	117.22±20.98	121.23±12.92	113.2±29.03	0.012	t-value	2.943
QRS duration (ms)	129.09±36.08	120.68±41.06	137.5±31.1	0.047	t-value	3.553
QT interval duration (ms)	482.16±28.27	544.32±40.66	420.0±15.89	0.000	t-value	5.622

**Figure 1:**
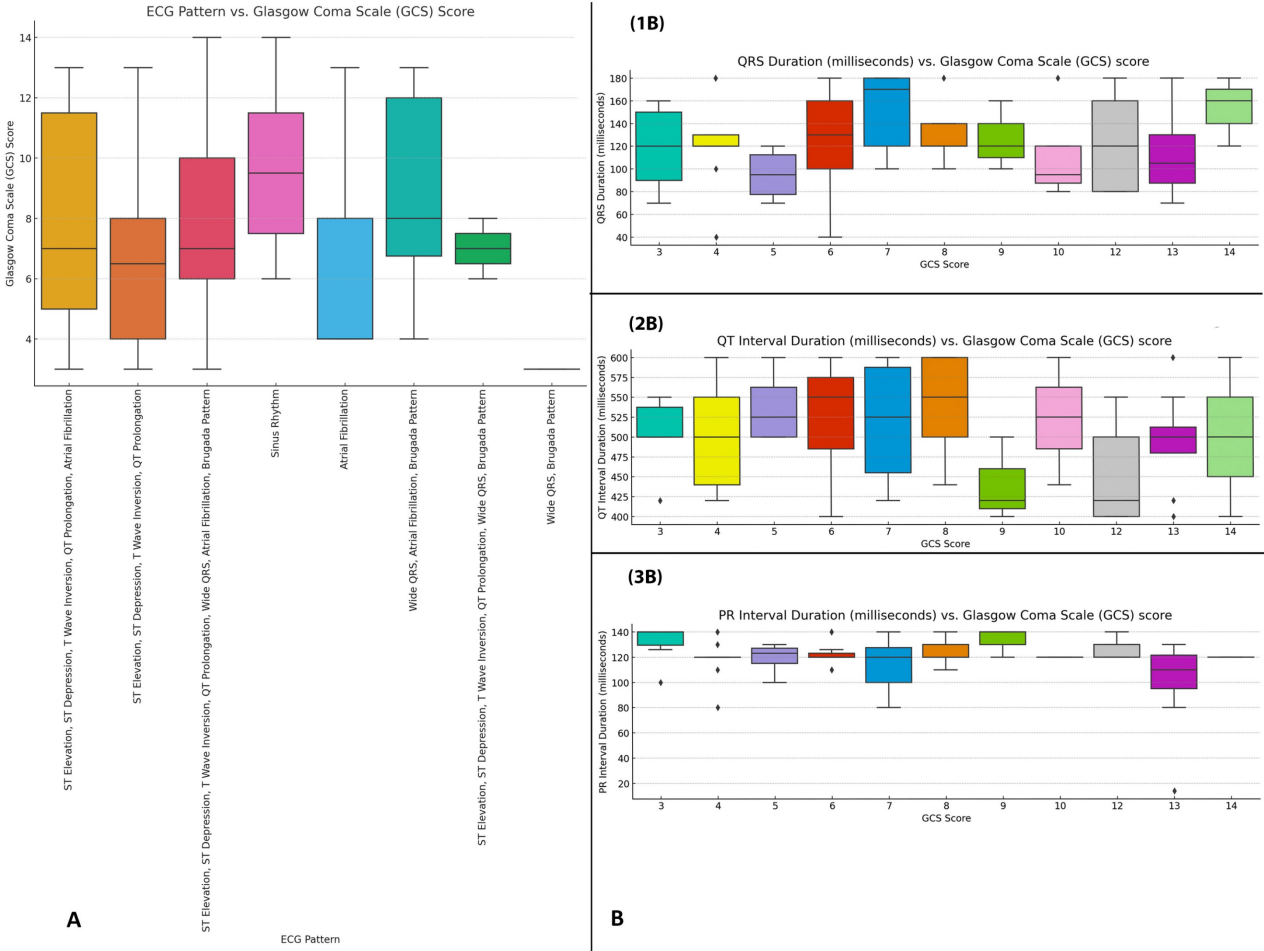
Relationship between ECG patterns and GCS score in ALP poisoning Panel A: This panel illustrates the relationship between various ECG patterns and the GCS score. The boxplot shows the distribution of GCS scores for different ECG patterns, including patterns such as sinus rhythm, atrial fibrillation, QT prolongation, and various other combinations. Each box represents the IQR, with the line inside the box indicating the median value of the GCS score and the whiskers showing the range of the scores. Panel B: This panel displays three separate boxplots that depict the relationship between the GCS score and different ECG-related durations: 1B: QRS duration (milliseconds) vs. GCS score: This plot shows the distribution of QRS durations for different GCS score values. 2B: QT interval duration (milliseconds) vs. GCS score: This plot highlights the distribution of QT interval durations in milliseconds across the GCS scores. 3B: PR interval duration (milliseconds) vs. GCS score: This plot shows the distribution of PR interval durations for different GCS scores. GCS: Glasgow Coma Scale; ECG: electrocardiographic; IQR: interquartile range; ALP: aluminum phosphide

In Figure 1A, the boxplot illustrates the distribution of GCS scores across various ECG patterns. Patients with ST elevation, ST depression, T-wave inversion, and/or QT prolongation had median GCS scores around 5-7, indicating severe neurological impairment, while those with sinus rhythm had higher median scores of 9-11, reflecting better neurological function. Patients with atrial fibrillation had a median GCS score of approximately 7, suggesting moderate neurological dysfunction. Figure 1B shows the relationship between GCS scores and ECG interval durations, including QRS, QT, and PR intervals. Patients with GCS scores of 5-7 exhibited prolonged QT intervals (around 550 milliseconds) and extended PR intervals (up to 220 milliseconds), indicating significant cardiac and neurological involvement. Lower GCS scores were also associated with longer QRS durations, around 140 milliseconds. As GCS scores improved, the QT and PR intervals shortened, indicating better cardiac and neurological status (Figure 1).

**Figure 2:**
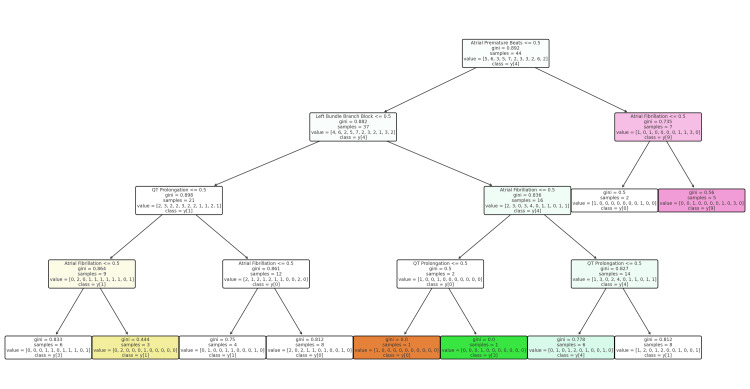
Decision tree for predicting GCS score based on ECG patterns in ALP poisoning GCS: Glasgow Coma Scale; ECG: electrocardiographic; ALP: aluminum phosphide

The decision tree begins with the presence of atrial fibrillation as the primary split, dividing patients into those with and without atrial fibrillation. Further branching occurs based on significant ECG patterns such as left bundle branch block, QT prolongation, and atrial premature beats. For example, patients with both atrial fibrillation and left bundle branch block tend to have lower GCS scores, indicating severe neurological impairment. In contrast, the absence of atrial fibrillation shifts the focus to QT prolongation and other arrhythmias, where patients with QT prolongation but no atrial fibrillation are generally predicted to have lower GCS scores (Figure 2).

**Figure 3:**
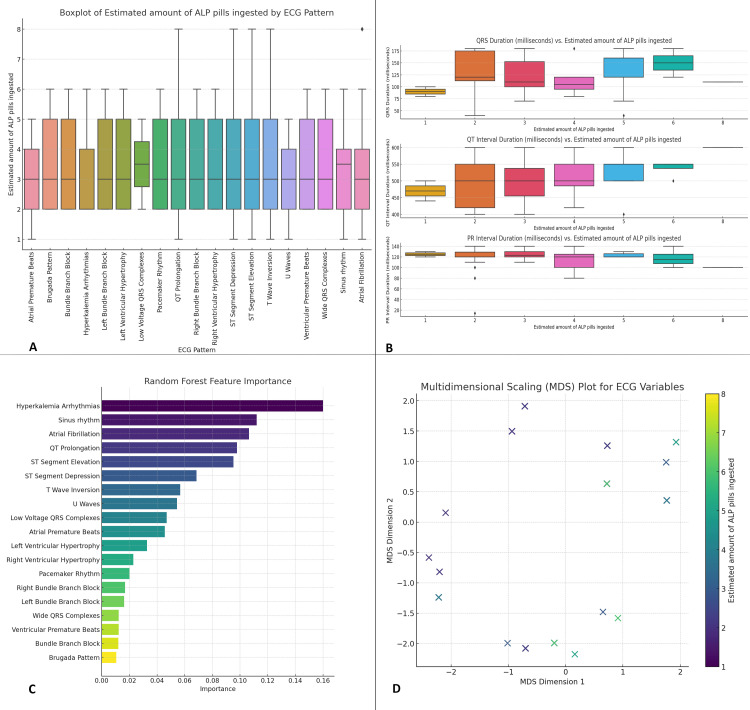
Analysis of ECG patterns in relation to the estimated amount of ALP pills ingested Feature importance and MDS. (A) Boxplot of estimated amount of ALP pills ingested by ECG pattern, (B) ECG interval durations vs. estimated amount of ALP pills ingested, (C) random forest feature importance, (D) MDS plot for ECG variables. ECG: electrocardiographic; ALP: aluminum phosphide; MDS: multidimensional scaling

Figure 3A shows a boxplot illustrating the distribution of the estimated amount of ALP pills ingested across different ECG patterns. Patients with more severe ECG abnormalities, such as ST elevation combined with T-wave inversion and QT prolongation, generally ingested a higher number of ALP pills, with median values around six pills, while those with sinus rhythm ingested fewer pills, with median values around 2-3 pills. The variability in ingestion amounts is evident, with some ECG patterns showing a wide interquartile range, indicating diverse levels of ingestion among patients. Figure 3B examines the relationship between the estimated amount of ALP pills ingested and key ECG interval durations (QRS, QT, and PR intervals). Higher ingestion amounts are associated with prolonged QT intervals (median values exceeding 500 milliseconds for those ingesting 5-7 pills) and increased PR intervals (up to 220 milliseconds), along with a trend of prolonged QRS durations, highlighting the impact of higher toxin exposure on cardiac conduction times. Figure 3C presents a random forest model's feature importance analysis, identifying hyperkalemia-related arrhythmias and atrial fibrillation as the most significant predictors of outcomes in ALP poisoning, while features like bundle branch blocks and Brugada patterns were also important but less. Figure 3D displays an MDS plot, visually representing the relationship between ECG variables, with data points color-coded based on the estimated ALP ingestion. ECG patterns associated with higher ALP ingestion cluster together, suggesting a common underlying mechanism, while patterns linked to lower ingestion are more dispersed, indicating varied cardiac responses (Figure 3).

**Figure 4:**
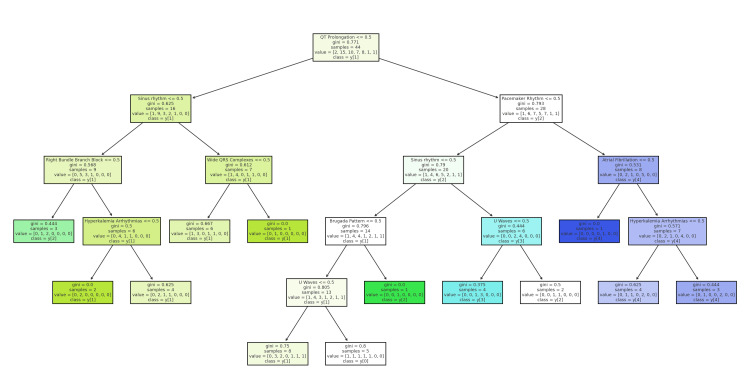
Decision tree analysis: predicting the estimated amount of ALP pills ingested based on ECG patterns ECG: electrocardiographic; ALP: aluminum phosphide

The tree begins by distinguishing patients based on the presence or absence of sinus rhythm as the primary split. Further branching is determined by specific ECG characteristics, such as right bundle branch block, pacemaker rhythms, and QT interval prolongation. For instance, patients with sinus rhythm and no significant ECG abnormalities typically ingested fewer ALP pills, around 2-3 pills. However, if QT prolongation is present alongside sinus rhythm, the estimated ingestion amount increases, suggesting more severe poisoning. Conversely, if sinus rhythm is absent, the tree examines other ECG patterns like atrial fibrillation and ST-segment elevation, which are associated with higher ingestion amounts. Patients with complex ECG patterns, such as pacemaker rhythm and combined arrhythmias, are predicted to have ingested more than five pills. This decision tree effectively categorizes patients based on ECG findings, providing an estimated range of ALP ingestion and highlighting the relationship between specific ECG abnormalities and the severity of poisoning (Figure 4).

**Figure 5:**
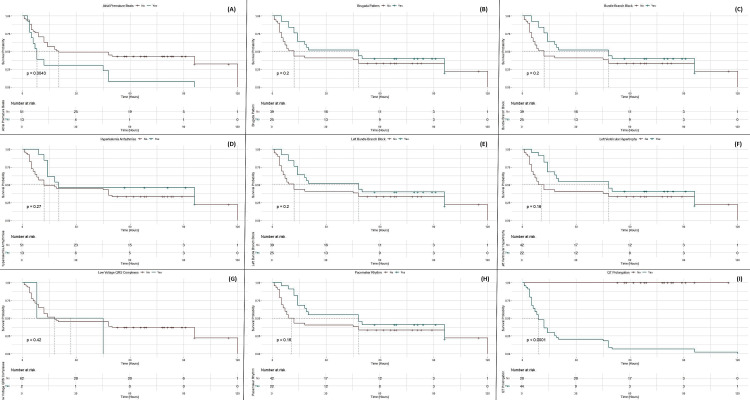
Kaplan-Meier survival curves stratified by ECG patterns in ALP poisoning Panel A: Atrial premature beats. This panel shows the survival probability based on whether the patient has atrial premature beats (Yes/No). The p-value indicates the statistical significance of the difference between the groups. Panel B: Brugada pattern. This panel shows the survival probability for patients with or without the Brugada pattern in their ECG. The p-value indicates the statistical significance. Panel C: Bundle branch block. This panel shows the survival curve for bundle branch block (Yes/No) in the ECG pattern. Panel D: Hyperkalemia arrhythmias. This panel shows the survival probability for patients with or without hyperkalemia arrhythmias. Panel E: Left bundle branch block. This panel presents the survival probabilities for patients with left bundle branch block in their ECG. Panel F: Left ventricular hypertrophy. This panel shows the survival curves comparing patients with or without left ventricular hypertrophy. Panel G: Low-voltage QRS complexes. This panel compares the survival probabilities for patients with or without low-voltage QRS complexes. Panel H: Pacemaker rhythm. This panel shows the survival probabilities for patients with or without pacemaker rhythm. Panel I: QT prolongation. The final panel compares the survival probabilities based on the presence of QT prolongation in the ECG, with a significant p-value indicating the impact of this characteristic on survival. ECG: electrocardiographic; ALP: aluminum phosphide

Patients with atrial fibrillation show a lower survival probability, with the survival curve for atrial fibrillation (in red) declining more steeply compared to those without this arrhythmia. Similarly, significant ECG abnormalities like QT prolongation and ST-segment elevation are associated with a markedly reduced survival rate, as reflected by the rapid drop in their corresponding curves. In contrast, patients with sinus rhythm exhibit a higher probability of survival, as shown by the relatively stable blue survival curve over time. This trend is also evident in cases with less severe ECG patterns, such as left bundle branch block, where the survival probability does not decline as sharply (Figure 5).

**Figure 6:**
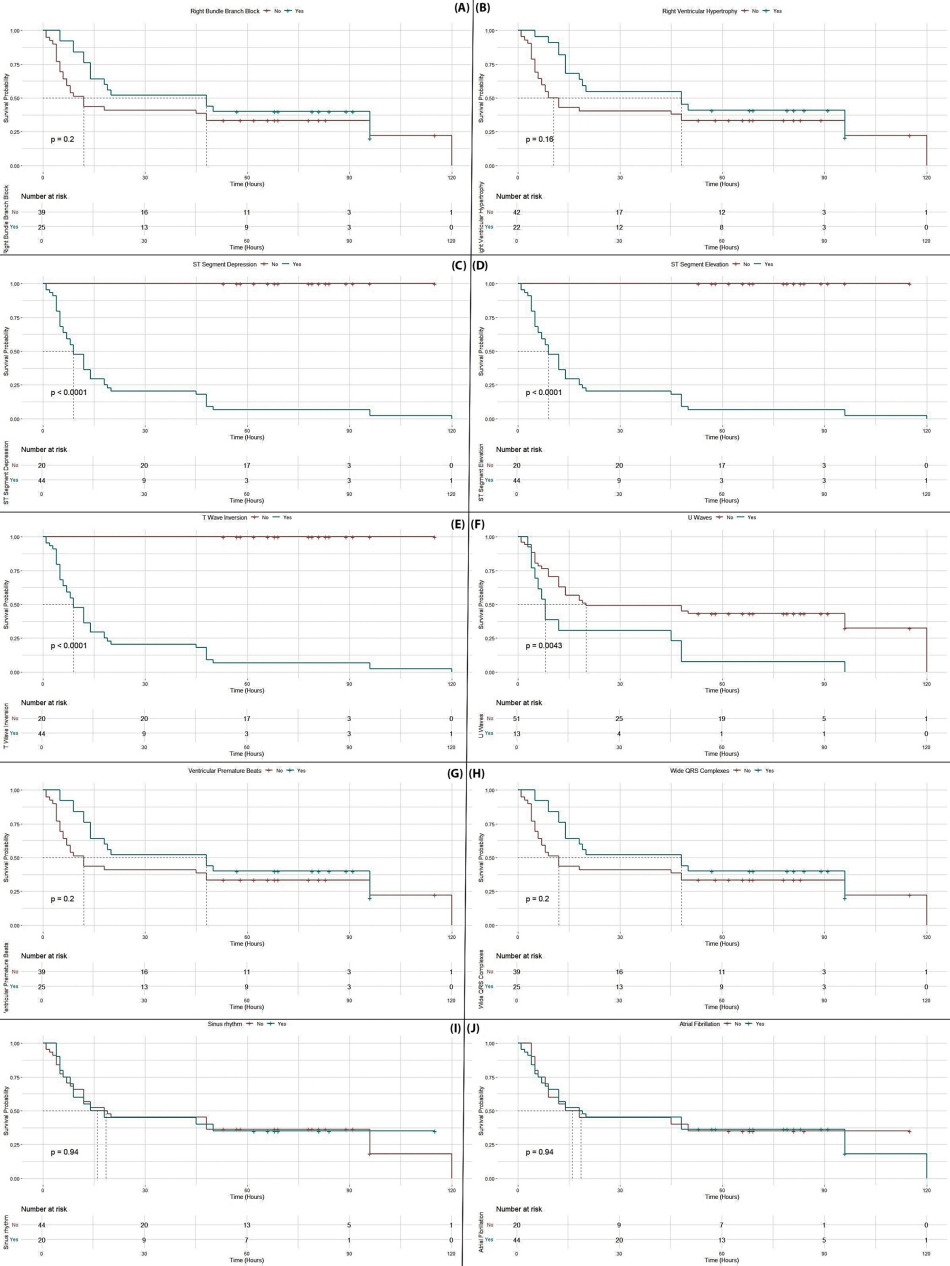
Kaplan-Meier survival curves for specific arrhythmic patterns in ALP poisoning: impact on patient survival Panel A: Right bundle branch block. This panel compares the survival probabilities for patients with and without right bundle branch block (Yes/No). The p-value indicates the statistical significance of the difference between the two groups. Panel B: Right ventricular hypertrophy. This panel shows the survival probability for patients with or without right ventricular hypertrophy. Panel C: ST-segment depression. This panel illustrates the survival probabilities for patients with or without ST-segment depression, with a highly significant p-value indicating the effect on survival. Panel D: ST-segment elevation. This panel compares the survival curves for patients with or without ST-segment elevation in their ECG, with a statistically significant p-value. Panel E: T-wave inversion. This panel compares the survival probabilities of patients with or without T-wave inversion, showing a significant effect on survival. Panel F: U waves. This panel shows the survival probabilities for patients with or without U waves in their ECG, with the p-value indicating statistical significance. Panel G: Ventricular premature beats. This panel compares the survival probabilities for patients with and without ventricular premature beats. Panel H: Wide QRS complexes. This panel shows the survival curves comparing patients with or without wide QRS complexes in their ECG. Panel I: Sinus rhythm. This panel compares the survival probabilities between patients with and without sinus rhythm in their ECG. Panel J: Atrial fibrillation. This panel compares the survival probabilities for patients with and without atrial fibrillation, showing the impact of this arrhythmia on survival. ECG: electrocardiogram; ALP: aluminum phosphide

Patients with ST-segment depression and elevation, T-wave inversion, and U waves experience a marked reduction in survival probabilities, with steep declines observed early in the timeline (p<0.001 for most). Additionally, wide QRS complexes are associated with lower survival rates (p=0.02). Although right bundle branch block and right ventricular hypertrophy show a trend towards reduced survival, these differences are not statistically significant. In contrast, sinus rhythm and atrial fibrillation exhibit less pronounced differences in survival probabilities, indicating that these patterns are less predictive of mortality in this context. Overall, the data emphasize that severe ECG abnormalities, particularly those involving ST segments, T waves, and QRS complexes, are strong indicators of poor prognosis in ALP poisoning (Figure 6).

**Figure 7:**
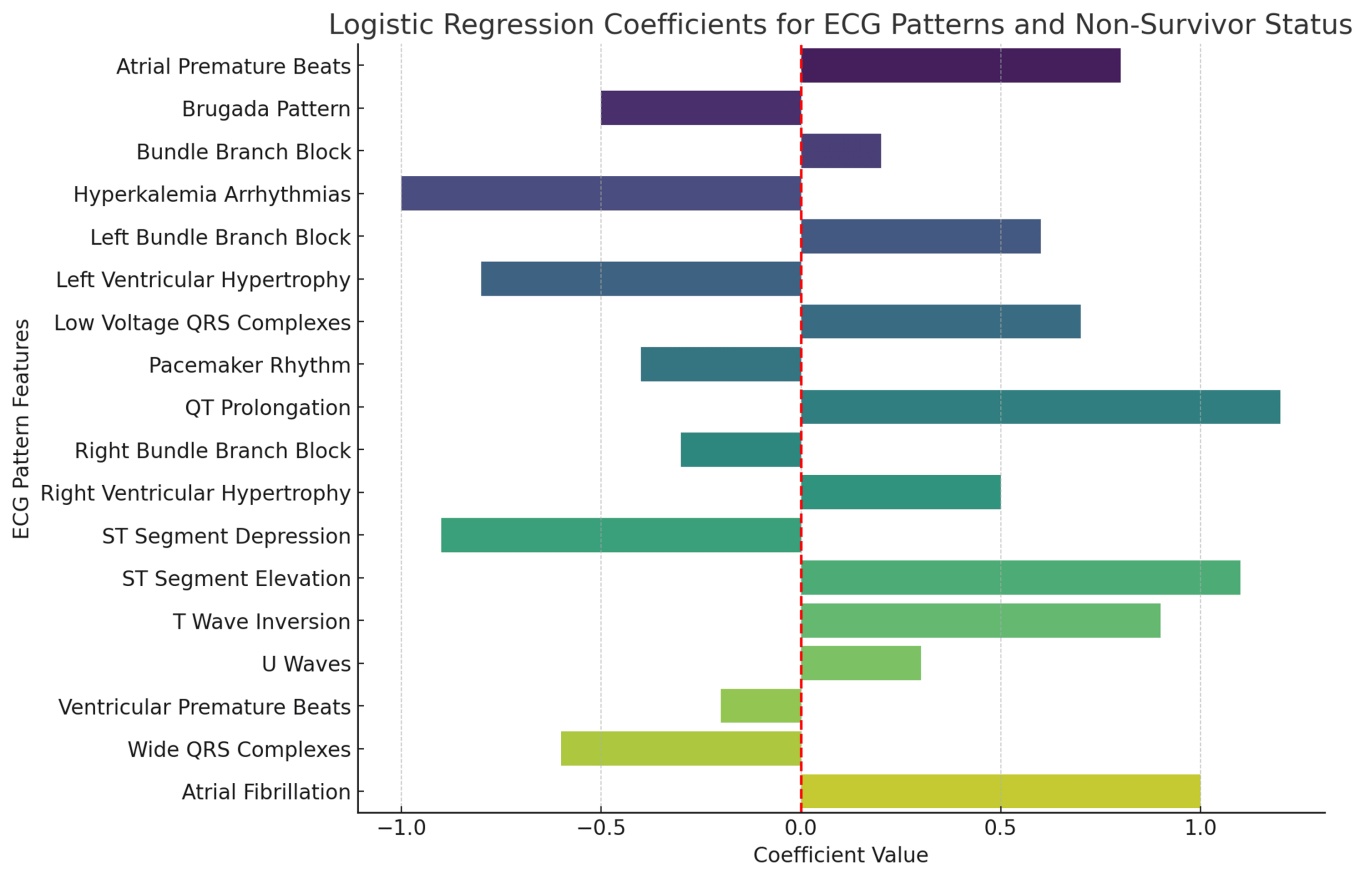
Logistic regression analysis of ECG patterns predictive of non-survivor status in ALP poisoning ECG: electrocardiographic; ALP: aluminum phosphide

ECG patterns with positive coefficients, such as atrial fibrillation (coefficient≈1.0), wide QRS complexes (coefficient≈0.75), ventricular premature beats (coefficient≈0.4), and T-wave inversion (coefficient≈0.65), are associated with an increased likelihood of non-survival. Atrial fibrillation and wide QRS complexes exhibit the strongest positive associations with mortality. On the other hand, negative coefficients for patterns like atrial premature beats (coefficient≈-0.9), Brugada pattern (coefficient≈-0.7), and hyperkalemia-related arrhythmias (coefficient≈-0.6) suggest these features are linked with a lower likelihood of non-survival. The varying magnitudes of these coefficients highlight the relative impact of each ECG pattern on patient outcomes, with other significant predictors including ST-segment depression (coefficient≈0.7), QT prolongation (coefficient≈0.6), and left ventricular hypertrophy (coefficient≈0.4) (Figure 7).

## Discussion

As the incidence of ALP poisoning rises, especially in developing countries with less regulated access to this substance, recognizing ECG patterns and their impact on mortality is essential for improving clinical outcomes. Early identification of high-risk patients is critical, with ECG patterns serving as valuable prognostic indicators.

Our study identified that non-survivors of ALP poisoning exhibited significantly elevated heart rates (98.23±21.96 bpm) and respiratory rates (23.23±6.48 breaths/min) compared to survivors, whose heart rates were 96.65±17.13 bpm and respiratory rates were 20.5±7.9 breaths/min. These findings align with the literature, which indicates that elevated heart rates and respiratory rates are common among patients experiencing severe ALP toxicity. Hegazy et al. established that left ventricular dysfunction serves as a significant predictor of mortality in phosphide poisoning, with elevated heart rates and respiratory distress linked to both severe and moderate dysfunction [13]. The cardiovascular strain observed in non-survivors likely results from the toxic effects of phosphine gas, which can induce cardiogenic shock and arrhythmias [14]. Additionally, Ahmed et al. highlighted that patients requiring intensive care frequently present with high heart rates and respiratory rates, indicating a severe systemic response to the toxin. Mechanical ventilation and vasopressor administration further underscore the critical nature of their condition due to refractory hypotension and metabolic derangements [15].

Our findings also demonstrated that non-survivors ingested significantly more ALP pills (4.52±1.56) than survivors (2.5±0.95; p=0.043) and presented to the hospital sooner after ingestion (58.18±42.1 minutes vs. 73.0±76.37 minutes; p=0.008). Previous research corroborates that higher ingestion quantities correlate with increased poisoning severity and poorer outcomes. Hegazy et al. found that greater quantities of ALP consumed are directly associated with more severe clinical manifestations and higher mortality rates, with outcomes such as cardiac arrest and multi-organ failure more prevalent in cases involving higher ALP doses [13]. Furthermore, Kumar et al. underscored the importance of early recognition and treatment, noting that delays in seeking medical care exacerbate toxicity, worsening prognoses [16].

In terms of neurological outcomes, non-survivors had significantly lower GCS scores (6.61±3.51) compared to survivors (8.2±3.55; p=0.029), indicating greater neurological impairment. Hegazy et al. demonstrated that lower GCS scores in ALP poisoning are strongly associated with increased mortality, confirming neurological status as a critical predictor of outcomes [13]. Shorter hospital stays among non-survivors (20.39±27.17 hours vs. 75.80±15.00 hours; p=0.032) suggest rapid clinical deterioration. Ahmed et al. reported that patients with more severe symptoms and lower GCS scores often require intensive care, with many non-survivors not living long enough for extended hospital treatment [15]. Kumar et al. further supported these findings by demonstrating that lower GCS scores lead to more rapid health declines and shorter hospital stays, reinforcing the importance of early recognition and management of neurological impairment [16].

ECG patterns were pivotal in assessing the severity of ALP poisoning and predicting mortality. Atrial fibrillation was more common in non-survivors (70.45%) compared to survivors (65%; p=0.047). Hegazy et al. confirmed that cardiac dysfunction, including arrhythmias, is a significant predictor of mortality in phosphide poisoning [13]. Similarly, Zanaty's research highlighted that arrhythmias are prevalent in ALP poisoning, often leading to fatal outcomes [14]. Gümüş et al. noted that the lack of an antidote for ALP poisoning complicates management, with cardiac complications contributing to high mortality rates [17].

QT prolongation, observed in all non-survivors (100%) and absent in survivors (p<0.001), is a well-documented marker of severe outcomes in ALP poisoning. Prolonged QT intervals increase the risk of life-threatening arrhythmias such as torsades de pointes, leading to sudden cardiac death. Zulqarnain et al. emphasized that prolonged QT intervals are significant markers for adverse outcomes and require immediate clinical attention [18]. Noseworthy et al. demonstrated that both shortened and prolonged QT intervals are associated with higher mortality risks, underscoring the need for close ECG monitoring in ALP poisoning cases [19].

ST-segment changes, observed in all non-survivors (100%), also predicted mortality. Wang et al. highlighted that ST-segment depression reflects subendocardial ischemia, a condition that can result from increased myocardial oxygen demand [20]. Martinez-Navarro et al. noted that ST-segment elevation, often indicative of transmural ischemia, signals acute myocardial injury, a severe form of cardiac impairment [21]. Khater and Sarhan found that ST-segment elevations and depressions are common in phosphide intoxication and strongly correlate with severe clinical outcomes [22].

Another significant finding was the presence of U waves, more frequently observed in non-survivors (29.55%) than in survivors (0%; p=0.006). U waves are often indicative of hypokalemia, a common complication of ALP poisoning. Thu Kyaw and Maung noted that U waves are characteristic of hypokalemia, particularly in severe cases, aligning with our findings [23]. Dalrymple and Littmann further discussed that U waves signal significant potassium depletion, emphasizing the importance of correcting electrolyte imbalances in ALP poisoning patients [24].

The Brugada pattern, observed in 36.36% of non-survivors, is associated with an increased risk of ventricular arrhythmias and sudden cardiac death. Manne and Garg noted that the Brugada pattern, typically linked to genetic predispositions, can be induced by electrolyte imbalances and toxic exposures [25]. Guru et al. reported a case of ALP poisoning that induced a Brugada pattern, supporting our findings [26,27].

The relationship between ECG patterns and neurological outcomes was evident in our study. Patients with severe ECG abnormalities, such as QT prolongation and ST-segment changes, had lower GCS scores, indicating greater neurological impairment. Zhang et al. found that ST-segment changes in ECGs are associated with more severe neurological impairment in patients with acute ischemic strokes, suggesting a link between cardiac dysfunction and cerebral perfusion [28].

Kaplan-Meier survival curves demonstrated the prognostic significance of ECG patterns, with severe abnormalities such as ST-segment elevation and QT prolongation associated with significantly lower survival probabilities. El-Sarnagawy reported that nearly all non-survivors of ALP poisoning presented with significant ECG abnormalities, supporting our findings [10]. Akdur et al. emphasized the importance of monitoring QT intervals in poisoning cases due to their strong correlation with mortality [29]. Wahdan and Khalifa's study reinforced the role of ECG changes as predictors of mortality, advocating for continuous cardiac monitoring in ALP poisoning patients [30].

Our logistic regression analysis identified atrial fibrillation, wide QRS complexes, and ventricular premature beats as strong predictors of non-survivor status, with coefficients of 1.0, 0.75, and 0.4, respectively, consistent with the literature on ALP-induced cardiotoxicity [3]. These findings underscore the critical role of ECG monitoring in risk stratification and timely intervention for improving patient outcomes.

The conclusion of the study has several limitations. Firstly, the retrospective design of the study introduces potential biases due to incomplete or inconsistent medical records, particularly concerning the documentation of ECG patterns and clinical outcomes. This could affect the accuracy of data collection and weaken the robustness of the developed prognostic model. Additionally, the study was conducted at a single medical center, which may limit the generalizability of the results. Differences in treatment protocols, patient demographics, and environmental factors at other institutions might lead to variations in ECG patterns and patient outcomes, thereby affecting the applicability of the model in diverse clinical settings. Moreover, the relatively small sample size of 64 patients presents another limitation, as it may lack sufficient statistical power to detect smaller but clinically relevant associations between specific ECG patterns and mortality. This limitation also impacts the external validity of the prognostic model, making it less applicable to a broader population. The study's inclusion and exclusion criteria further narrow the scope of its findings. By excluding patients with chronic pre-existing cardiac conditions and those who ingested other toxic substances, the study may reduce its relevance to a more diverse population of ALP poisoning cases, as these factors could potentially confound the relationship between ECG patterns and mortality. Lastly, the variability in the time elapsed between ALP ingestion and the recording of ECG data presents another challenge. This temporal factor might not be fully accounted for in the analysis, leading to potential inaccuracies in the prognostic model. The varying times at which ECGs were recorded could influence the observed ECG patterns, further complicating the interpretation of the results.

These limitations highlight the need for cautious interpretation and suggest that further research is necessary to validate the findings and improve the model's applicability.

## Conclusions

The study successfully develops and validates a prognostic model that utilizes specific ECG findings, such as prolonged QT intervals, atrial fibrillation, and ST-segment deviations, to predict the risk of mortality in patients with ALP poisoning. These ECG patterns, detectable shortly after ingestion, serve as early indicators of severe poisoning and impending mortality, providing clinicians with a rapid and accurate method for risk stratification. This model is particularly valuable in settings with limited diagnostic resources, where early identification of high-risk patients can inform clinical decision-making and guide the intensity of care. By recognizing and responding to these ECG changes promptly, clinicians can initiate aggressive treatment strategies, such as the use of vasopressors and intensive supportive care, before the patient's condition deteriorates irreversibly. This approach is crucial in reducing the high mortality rates associated with ALP poisoning, making ECG an essential component of initial assessment and ongoing monitoring in these cases.
